# Resting state EEG in young children with Tuberous Sclerosis Complex: associations with medications and seizures

**DOI:** 10.1186/s11689-025-09590-z

**Published:** 2025-01-18

**Authors:** Caitlin C. Clements, Anne-Michelle Engelstad, Carol L. Wilkinson, Carly Hyde, Megan Hartney, Alexandra Simmons, Helen Tager-Flusberg, Shafali Jeste, Charles A. Nelson

**Affiliations:** 1 Department of Psychology, University of Notre Dame, 340 Corbett Family Hall Notre Dame, South Bend, IN 46556 USA; 2 Laboratories of Cognitive Neuroscience, Division of Developmental Medicine, Boston Children’s Hospital, Brookline, MA USA; 3 Graduate School of Education, Harvard University, Cambridge, MA USA; 4 Department of Pediatrics, Harvard Medical School, Boston, MA USA; 5 School of Public Health, UCLA, Los Angeles, CA USA; 6 Department of Psychological & Brain Sciences, Boston University, Boston, MA USA; 7 Department of Neurology, Children’s Hospital LA, Los Angeles, CA USA

**Keywords:** Tuberous Sclerosis Complex, Seizures, Epilepsy, EEG, GABA agonists, Biomarker

## Abstract

**Background:**

Tuberous Sclerosis Complex (TSC) is a rare genetic condition caused by mutation to *TSC1* or *TSC2* genes, with a population prevalence of 1/7000 births. TSC manifests behaviorally with features of autism, epilepsy, and intellectual disability. Resting state electroencephalography (EEG) offers a window into neural oscillatory activity and may serve as an intermediate biomarker between gene expression and behavioral manifestations. Such a biomarker could be useful in clinical trials as an endpoint or predictor of treatment response. However, seizures and antiepileptic medications also affect resting neural oscillatory activity and could undermine the utility of resting state EEG features as biomarkers in neurodevelopmental disorders such as TSC.

**Methods:**

This paper compares resting state EEG features in a cross-sectional cohort of young children with TSC (*n* = 49, ages 12–37 months) to 49 age- and sex-matched typically developing controls. Within children with TSC, associations were examined between resting state EEG features, seizure severity composite score, and use of GABA agonists.

**Results:**

Compared to matched typically developing children, children with TSC showed significantly greater beta power in permutation cluster analyses. Children with TSC also showed significantly greater aperiodic offset (reflecting nonoscillatory neuronal firing) after power spectra were parameterized using SpecParam into aperiodic and periodic components. Within children with TSC, both greater seizure severity and use of GABAergic antiepileptic medication were significantly and independently associated with increased periodic peak beta power.

**Conclusions:**

The elevated peak beta power observed in children with TSC compared to matched typically developing controls may be driven by both seizures and GABA agonist use. It is recommended to collect seizure and medication data alongside EEG data for clinical trials. These results highlight the challenge of using resting state EEG features as biomarkers in trials with neurodevelopmental disabilities when epilepsy and anti-epileptic medication are common.

**Supplementary Information:**

The online version contains supplementary material available at 10.1186/s11689-025-09590-z.

## Background

Electroencephalography (EEG) offers a low-cost, noninvasive neuroimaging method that is feasible in people of all ages and abilities. As such, EEG holds promise as a potential tool to examine brain-based biomarkers for prediction of treatment response for neurodevelopmental disabilities. EEG-based biomarkers could be particularly useful as an endpoint in clinical trials in rare genetic syndromes; for these conditions, diagnostic biomarkers already exist (i.e., genetic testing) but biomarkers of treatment response or prognostication remain elusive. Many children with neurodevelopmental disabilities such as Tuberous Sclerosis Complex (TSC) experience seizures [1] and may be prescribed EEG-altering antiepileptic medications. However, seizures and antiepileptic medications may limit the reliability of resting EEG biomarkers. We must understand the impact of seizures and antiepileptic medication on the EEG power spectrum given current interest in EEG-based biomarkers as clinical trial endpoints for neurodevelopmental disabilities like TSC, autism, and others [2–5]. The lessons learned from TSC in the search for EEG-based biomarkers might extend to other neurodevelopmental disabilities as well, since there is a high prevalence of seizures in many neurodevelopmental disabilities such as Rett Syndrome (60–80% [6]), Fragile X Syndrome (20% [1]), 22q11.2 Deletion Syndrome (11% [7]), CDKL5 Deficiency Disorder (nearly 100% [1]), autism (12% [8]) and others.

### Tuberous Sclerosis Complex

Tuberous Sclerosis Complex (TSC) is a rare autosomal dominant disorder that results from mutations in the *TSC1* or *TSC2* genes. TSC has a prevalence of approximately 1 in 7,000 births and is usually detected in utero or the first year of life [9]. The inactivation of *TSC1/TSC2* leads to overactivation of the mammalian target of rapamycin (mTOR) pathway, which in turn causes unchecked cell growth and proliferation in some regions of the body, particularly the heart, kidneys, skin, and brain. In the brain, individuals with TSC show hamartomas, including cortical tubers, that impact neuronal function and connectivity [10]. Most children with TSC have epilepsy (70–80% [11]) and co-occurring autism (between 46 and 66% [12]). Families increasingly report that their greatest concerns are the TSC-associated neuropsychiatric disorders (TAND) that many individuals with TSC exhibit, such as autism and intellectual disability.

Previous EEG research in infants and toddlers with TSC has identified reduced maturity and connectivity in the EEG power spectrum [13, 14], and some of these differences have predicted later cognitive development and autistic features [13, 15]. Studies of older children, adolescents, and adults have reported alterations in connectivity [16–18] and task-based differences [19, 20]. In a younger cross-sectional sample of 10 toddlers with TSC and 12 typically developing children ages 18–30 months, Stamoulis et al. (2015) noted possible delayed maturation as indexed by a developmental shift of the dominant high-frequency spectral content that occurred later in children with TSC compared to typically developing controls. Children with TSC maintained higher frequencies at older ages, with non-random EEG components present in the high gamma (> 50 Hz) and ripple (> 80 Hz) frequencies [14]. De Ridder et al. (2020) also reported early dysmaturity in a clinical sample from the EPISTOP study using a different proxy of maturity. Using neonatal EEGs and 24-month developmental assessment data in 64 children with TSC, De Ridder and colleagues reported that more dysmaturity (indexed by power, range EEG, entropy, and Hurst exponent) predicted more autism traits, as well as lower cognitive, language, and motor developmental scores at 24 months [13]. In a similar vein, Dickinson et al. (2019) examined neural network development via features of alpha band oscillations (alpha power, peak alpha frequency, and alpha phase coherence) in a longitudinal sample from 12 to 36 months of 35 toddlers with TSC and 20 typically developing toddlers. Toddlers with TSC showed reduced interhemispheric alpha phase coherence at 12 and 24 months, and the difference was more pronounced at 24 months in TSC toddlers later diagnosed with autism. Peak alpha frequency at 24 months predicted 36 month nonverbal and verbal cognition in both TSC and typically developing children [15]. These prior studies provide valuable knowledge about neurophysiological differences in an understudied population, yet the very different EEG metrics used across studies make it difficult to compare or synthesize findings. Finally, despite the high prevalence of epilepsy and antiepileptic medication use in individuals with TSC, none of the extant literature describing EEG findings in TSC have accounted for the potential impact of antiepileptic medication.

### Effects of seizures and medications on EEG

Seizures affect the power spectrum in a number of ways depending on seizure type [21, 22]. For example, children who experience infantile spasms, a common early seizure type in TSC, show increased EEG amplitude and spectral power across all frequency bands between seizure periods [23]. Focal seizures, the second predominant seizure type in TSC, lead to increased relative power in beta and gamma bands [24], and gamma oscillations may be observed hours before seizure onset and correspond to where a focal seizure will originate [25]. In addition, seizure medications influence EEG activity and the power spectrum. GABA agonists (e.g., vigabatrin) are commonly prescribed to control seizures by increasing inhibitory activity [26], and in both humans and animal models, GABA agonists have consistently been associated with increased beta power [27–30]. In pharmacological studies of healthy adults, the administration of GABA agonists appears to consistently increase beta power [31–33], but results differ on whether peak beta frequency is unaltered [31, 32] or decreased with the spectral peak widened [34].

### Periodic and aperiodic powers

**Fig. 1:**
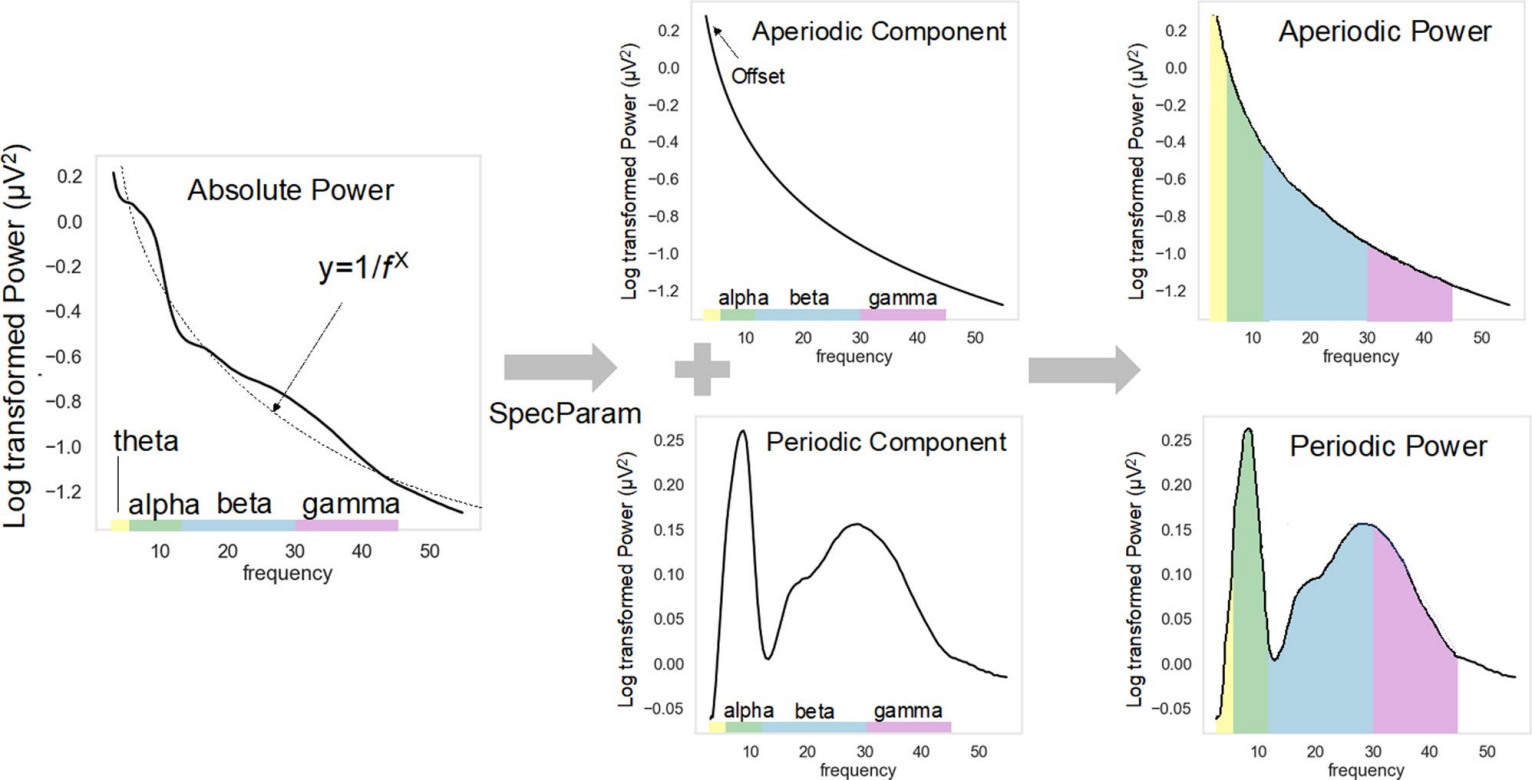
Decomposition of absolute spectral power into periodic and aperiodic components. The absolute power spectrum can be decomposed into the periodic and aperiodic components by fitting an exponential decay curve (y = 1/f) to the absolute power spectrum to model the aperiodic component. The 1/f curve (aperiodic component) can be described with the offset value (similar to intercept) and exponent (reflects how steep or shallow the curve is, similar to slope). The modeled 1/f curve can then be subtracted from the absolute power spectrum, leaving only the periodic, oscillatory curve. This decomposition can be implemented using the SpecParam algorithm [36] (also known as FOOOF v 1.0.0 [35])

Prior studies investigating the EEG power spectra in TSC have largely focused on absolute or relative power. However, parametrization of the power spectrum into aperiodic and periodic components can provide a more accurate estimate of non-oscillatory and oscillatory activity [35, 36], and may be valuable in considering differences in excitatory/inhibitory balance and the impact of seizure medications on beta oscillations in TSC (Fig. 1). The aperiodic component of the power spectra is defined by the 1/f power law distribution of the absolute power spectra. The aperiodic component can be described by an offset and slope (defined as the χ in the 1/f^χ^ formula), with the offset thought to reflect broadband, non-rhythmic firing [37, 38], and the slope hypothesized to reflect the state of excitatory-inhibitory (E/I) balance in the network. Studies from human, animal, and computational models suggest that a flatter (reduced) slope is associated with increased excitation over inhibition [39–41]. The periodic component, defined by the portion of the absolute spectrum rising above the aperiodic component, reflects oscillatory activity in narrow frequency bands [35, 42]. As there are individual differences in both the aperiodic and periodic components, parametrization of each can provide a more accurate measurement of peak amplitude and peak frequency of different narrow band oscillations [35, 36, 42].

### This study

In order to explore the feasibility of resting EEG as a biomarker in neurodevelopmental disabilities like TSC, the present study aims to first characterize the resting state EEG power in toddlers with TSC against age- and sex-matched typically developing children. Specifically, we present analyses using absolute, aperiodic, and periodic power that allow for comparison to prior studies that reported findings regarding absolute EEG power or maturity in young children with TSC. Second, we leverage seizure frequency and medication information to quantify the effects of seizure and medications on the TSC resting state power spectrum. We hypothesize that GABAergic medications will be associated with increased inhibitory activity in children with TSC as reflected by increased beta power. We hypothesize that the effects of seizures will be confounded and overshadowed by GABAergic medication use, as prior research has shown that both increases in GABA receptors and epileptic activity are associated with increases in beta power.

## Methods

### Participants

Young children with TSC were recruited into a multisite randomized control trial (JETS: NCT03422367) of a behavioral intervention to target social communication skills (JASPER: Joint Attention, Symbolic Play, Engagement & Regulation [43]) between 2017 and 2023. Children traveled to one of the two participating sites (Boston Children’s Hospital or UCLA Health) for a two-day baseline in-person assessment that included clinical characterization and EEG data collection. This manuscript reports baseline assessment data only.

Children between ages 12 and 56 months with a clinical diagnosis of TSC diagnosis were eligible for the study, and 57 provided resting state EEG recordings. Seven individuals with TSC were excluded because they did not have EEG recordings that met quality control thresholds (see below), and one individual with TSC was excluded from this analysis to facilitate matching with a typically developing control cohort. The final sample included 49 children. The age range of children included in this cross-sectional analysis was 12–37 months (M = 22.2(7.4) months; Table 1). Consistent with the literature, 40.8% of TSC participants were reported to have experienced at least one seizure in the last month, 67.4% currently or previously experienced infantile spasms, and 96% were taking at least one medication (M = 2.4(1.4) medication). MRI and tuber location were not available.

**Table 1:** Participant characteristics

	Tuberous Sclerosis Complex Participants	Typically developing controls
N	49	49
Age, months, mean(SD)	22.2 (7.4)	23.0 (8.1)
Sex, % male	51.0%	51.0%
Mullen Verbal Developmental Quotient, mean(SD)	63.2 (23.6)	117.9 (17.8)
Mullen Nonverbal Developmental Quotient, mean(SD)	73.1 (22.1)	114.8 (14.2)
**Seizures**		
% current seizures (last 2 months)	40.8%	
% on GABA antagonist	85.7%	
% infantile spasms: current ⦁ past ⦁ no history of spasms	8.2 ⦁ 59.2 ⦁ 32.7%	
Number of seizure medications	2.4 (1.4)	
Surgery	8.2%	

Comparison data from typically developing (TD) children was drawn from the Infant Screening Project 2 (ISP2), a longitudinal study of development that was conducted at Boston Children’s Hospital and Boston University from 2015 to 2020 (IRB P00018377). Developmental and EEG data were collected longitudinally at 12, 18, 24, and 36 months. Data from one timepoint per child were drawn from the ISP2 dataset to create a cross-sectional cohort of typically developing children matched 1:1 on age and sex with the TSC cohort. Children selected for the typically developing comparison cohort met the following criteria: no developmental delay confirmed by parent report and/or scores on standardized developmental assessments (e.g., Vineland, Mullen Scales of Early Learning), no history of seizures, no first degree relative with autism spectrum disorder, birthweight > 5.5 pounds, gestational age ≥36 weeks, and no genetic or neurological condition.

### Measures

#### Clinical

**Fig. 2:**
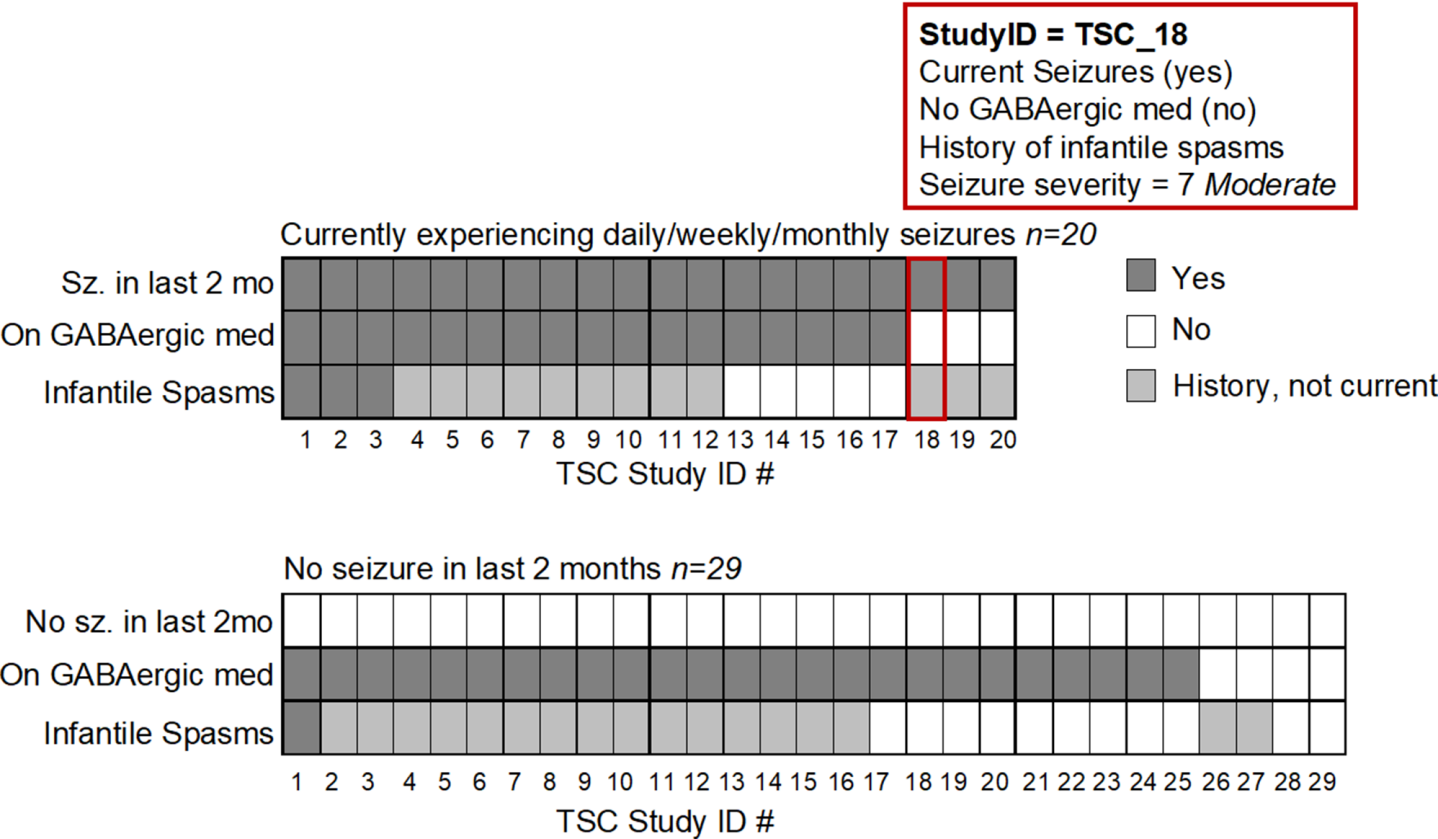
Seizure frequency, medication, and infantile spasms in individual TSC participants. Data reflect 49 participants with TSC in a randomized control trial of the JASPER behavioral intervention. At baseline, participants presented with heterogenous profiles of seizure frequency, GABAergic medication use, and presence of infantile spasms. The profile of each participant is depicted as a column of three shaded rectangles reflecting the presence (dark gray), absence (white), or history (light gray) of each clinical feature. For example, participant #18 outlined in red was reported at baseline to experience seizures at least monthly over the last two months; not to take a GABAergic medication; and to have a history of infantile spasms but not currently experience infantile spasms. Seizure severity scores are derived from the E-Chess [45] and incorporate the frequency of seizures, types of seizures (including infantile spasms), and number of anti-epileptic mediations (all classes)

Seizure, infantile spasm, and medication data were collected from parent report at baseline (Fig. 2). Since GABAergic medications are known to cause increased beta power [44], seizure medications were classified by mechanism of action into GABAergic medications and non-GABAergic medications (Table S1). As children with TSC have varying degrees of seizure activity, a composite seizure severity score was created using the E-Chess [45] to integrate frequency, medications, and types of seizures according to parent report. The seizure severity score ranged from 0 to 12 and reflected the sum of three variables: frequency of seizures in the last two months (0 = no seizures, 4 = more than daily seizures); number of current anti-epileptic medications (range 0–6); and total number of seizure types reported (1 point per type of seizure such as generalized, drop seizures, infantile spasms, etc.; range 0–5). Seizure severity scores were classified as low (0–2), moderate (3-7), or high (8-12).

The Mullen Scales of Early Learning (MSEL) was administered to assess developmental level. In order to avoid floor effects, Developmental Quotients (DQ) for each subscale were calculated (age equivalent / chronological age), then subscales were averaged to create a nonverbal DQ (visual reception + fine motor) and a verbal DQ (expressive language + receptive language). Autism diagnoses were not reliably available at the baseline timepoint given the age range of this cross-sectional cohort (35% of sample under 18 months).

#### EEG recording

Resting state EEG data were collected continuously for at least 2 min at UCLA or Boston Children’s Hospital in a dimly light, electrically-shielded, sound-attenuated room. The child sat on a caregiver’s lap and watched a screensaver-style video of bubbles. EEG data were recorded using a 128-channel Hydrocel Geodesic Sensor Net (EGI, Inc., Eugene, OR, USA) that contains sponge-based carbon fiber electrodes. The sponges were first soaked in a solution of 6mL KCl/L of water and 5 mL of baby shampoo to facilitate conductance, then the net was placed over the child’s head, and electrodes were carefully seated on the scalp with impedances under 100 ohms. The net was connected to a DC-coupled amplifier (Net Amps 300 amplifier EGI) at a sampling rate of 500 samples per second, and referenced online to the vertex electrode (Cz). Similar data recording protocols and preprocessing pipelines were used for the matched typically developing cohort.

#### EEG preprocessing

Raw EEG data were collected in NetStation (Magstim EGI) and exported to MATLAB (version 2021b) for processing using the Batch EEG Automated Processing Platform (BEAPP version 4.1 [46]) with embedded Harvard Automated Preprocessing Pipeline for EEG (HAPPE [47]).

These automated processing pipelines were chosen over visual inspection methods for several reasons. First, automated pipelines have the advantage of removing subjectivity/human error and improving reproducibility of analyses. HAPPE has been shown to improve data retention while successfully removing artifact when compared to alternative approaches, including manual segment rejection and ICA alone (see [47] for a report of comparisons). Finally, after EEGs are processed, the BEAPP/HAPPE pipeline provides measures of data quality so that researchers can make informed decisions about data rejection.

Briefly, each EEG underwent a 1 Hz high-pass and 100 Hz low-pass filter, downsampling to 250 Hz, removal of 60 Hz line noise, then artifact detection and bad channel rejection using wavelet-enhanced independent component analysis (ICA) and the Multiple Artifact Rejection Algorithm (MARA; [48]). Given the short length of the recording (2 min) and the use of high-density nets (128-channels), a subset of electrodes was included in this processing. Constraining the number of components in ICA decomposition is necessary to ensure robust and stable results. This is done by reducing the number of channels based on available EEG data length and sampling rate to provide appropriate data samples for reliable and stable ICA decomposition [47, 49]. A total of 37 channels were used. In addition to 10–20 electrodes, the following electrodes were included in processing for all participants: 4, 19, 13, 112, 55, 67, 77, 28, 117, 47, 98, 75, 65, 90, 37, 87, 41, 103. Following ICA artifact detection and removal of bad channels, data were re-referenced using the average across channels, and then detrended to the signal mean. Processed data were segmented into 2-second segments, and segments with retained artifact were rejected based on HAPPE’s amplitude and joint probability criteria using a 40 µV amplitude limit. Files were retained that met the following criteria, modeled after studies analyzing similar developmental populations [47, 50]: participants had 20 or more good segments (*n* = 5 excluded), > 80% good channels, mean or median retained artifact probability < 0.3 (*n* = 2 excluded), percent independent components rejected as artifact < 80%, and percent variance retained after artifact rejection > 25%.

#### EEG power analysis and parameterization

Using BEAPP software in MATLAB [46], power spectral densities for each electrode were estimated using multitaper spectral estimation with three orthogonal tapers. The power at each electrode was calculated for each frequency bin (0.5 Hz frequency resolution) for each two-second segment, then averaged across all segments for that electrode and frequency bin.

The power spectra were then separated into periodic and aperiodic components using SpecParam v1.0.0 (also known as FOOOF [35, 36]) in the fixed mode (no spectral knee) from 2 to 55 Hz. Settings for the algorithm were as follows: peak width limits: 0.5–18.0; max number of peaks: 7; and peak threshold: 2. To evaluate the periodic component, the SpecParam estimated aperiodic component was subtracted from the absolute power spectrum.

#### Extraction of EEG features

After parameterization into aperiodic and periodic spectra, features of the EEG were extracted for analysis. Aperiodic offset and slope were provided by SpecParam. Power was computed for the three spectral density curves (absolute, aperiodic and periodic power spectrum) using the integral under the curve for the following frequency ranges: theta (4–5 Hz), alpha (6-11 Hz), low beta (12-19 Hz), high beta (20–29 Hz), broad beta (12–29 Hz), and gamma (30–44 Hz). Next, peaks were identified within the broad alpha and broad beta ranges by identifying, for each child, the local maximum within the designated frequency range. The power value and frequency value at that peak were normalized with a log10 transformation (log10(uV^2)/Hz). Based on past literature [15], two regions of interest (Fig S1) were analyzed for all features: frontal (electrodes 24, 124, 11, 28, 117, 19, 4) and posterior (electrodes 70, 75, 83, 67, 77).

### Statistical analyses

1. Comparison of the power spectrum in TSC to typical development.

To identify differences in the power spectral density between the TSC and TD groups across the frequency range (2–44 Hz) for both full spectral power and periodic power, cluster permutation testing (*n* = 1000 permutations, 2-tailed, threshold for forming a cluster *p* < 0.05) with a cluster-level significance threshold of *p* < 0.05 was implemented via the MNE software package in Python (v3.8.5) Jupyter Notebooks (v2.2.6).

To identify group differences in aperiodic components (frontal and posterior intercepts and slopes), logistic regressions were conducted to predict group status (TD vs. TSC), implemented in RStudio (4.0.3).

2. Seizures and medication use within the TSC cohort.

The TSC cohort was stratified by low (0–2),moderate (3-7), and high (8-12)  seizure severity composites (integrating frequency, number of medications, and seizure type), and by use of GABAergic medications, which are known to affect spectral beta power. A two-way ANOVA was conducted to test the main effects and interaction of GABAergic medications and seizure severity on frontal beta peak. Post hoc comparisons were conducted and adjusted for multiple comparisons using Tukey’s HSD implemented in RStudio (4.0.3).

$$\begin{aligned}frontal\:beta\:peak\:amplitude=&{\beta\:}_{0}+{\beta\:}_{1}Seizure\:Severity\\&+{\beta\:}_{2}GABA+{\beta\:}_{3}Seizure\:Severity\\&\times\:GABA+\varepsilon\end{aligned}$$

## Results

### Children with TSC show greater beta power and greater aperiodic offset than typically developing controls

**Fig. 3:**
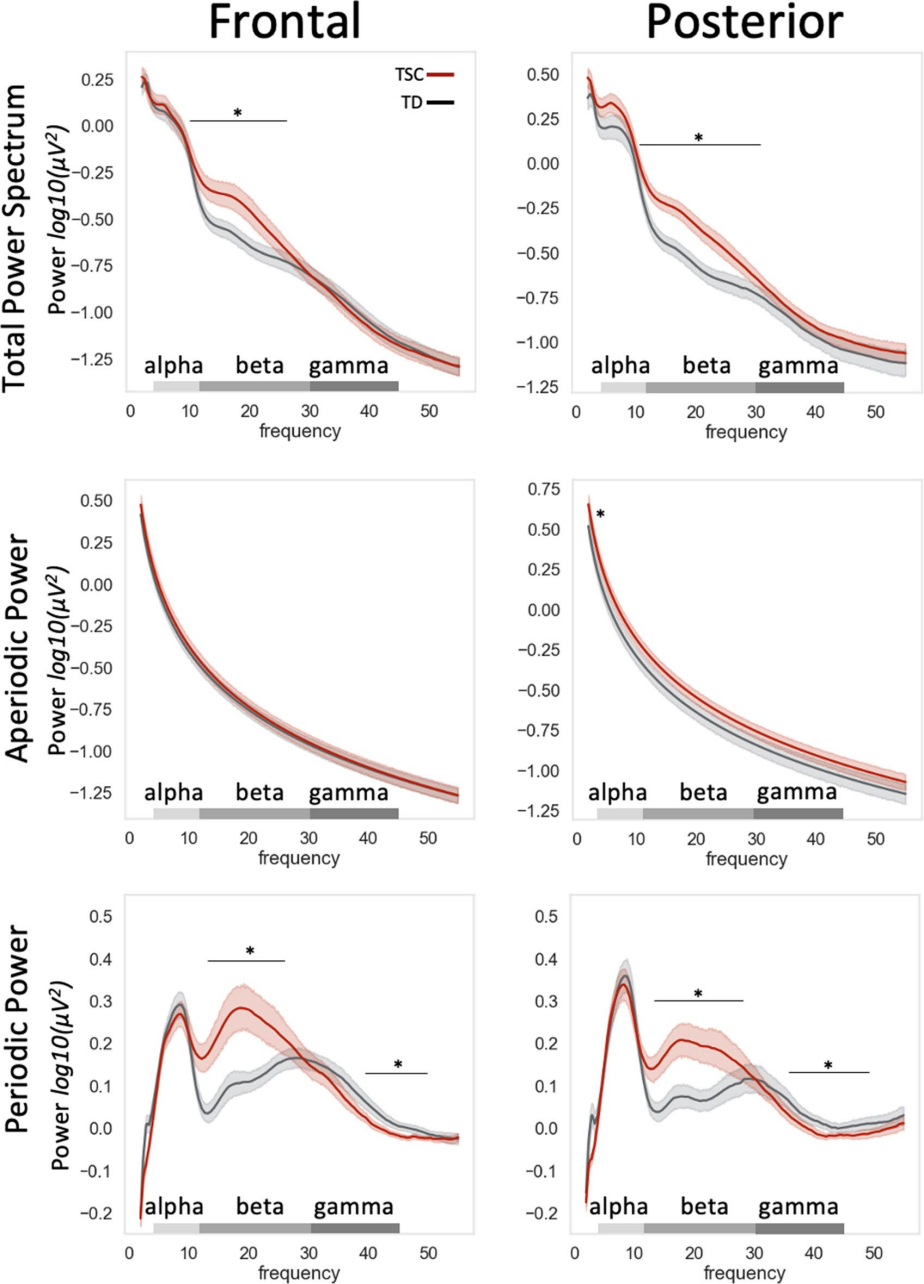
TSC and Typical Development parameterized power spectra. TSC and TD groups showed significantly different resting power in several frequency ranges, denoted by an asterisk and black bar spanning the significant frequency range. In aperiodic power (middle row), the TSC group showed significantly greater broadband nonscillatory neuronal firing than the TD group in the posterior intercept, denoted by an asterisk. No group differences were observed in the slope, which reflects the excitatory-inhibitory balance

**Fig. 4:**
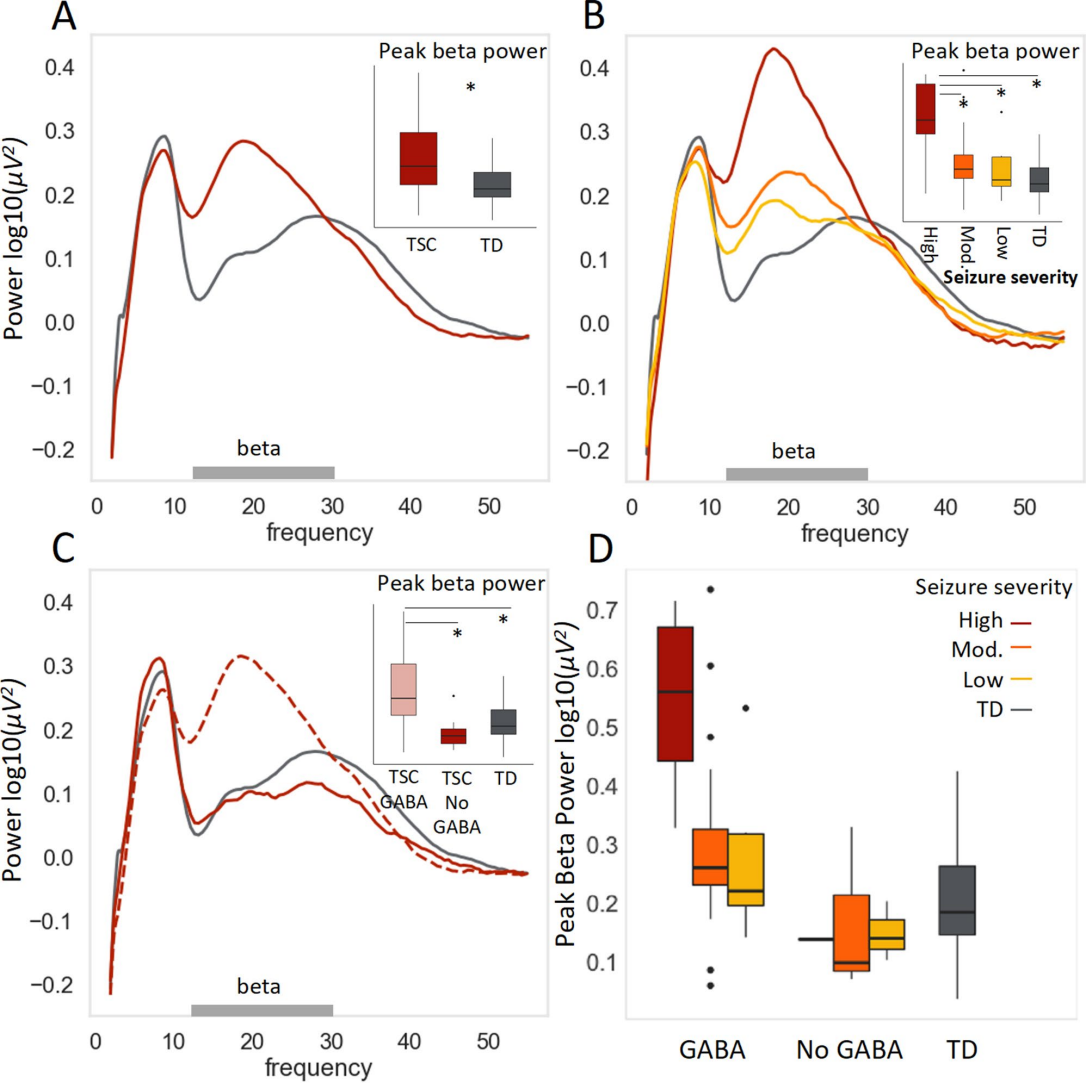
Parameterized frontal periodic power spectrum stratified by seizure composite and GABAergic medication use. (**A**) As a group, children with TSC (red, *n* = 49) showed significantly greater peak beta power than age- and sex- matched typically developing controls (gray, *n* = 49). (**B**) When the TSC participants were stratified by seizure severity composite, seizure severity appeared to drive the elevated peak beta power finding. The high seizure severity group (red, *n* = 13) showed significantly greater peak beta power than the moderate (orange, *n* = 23) and low (yellow, *n* = 12) seizure severity groups, neither of which differed significantly from typically developing controls (gray, *n* = 49). (**C**) When participants with TSC were stratified by GABAergic medication use, GABAergic medication use also appeared to drive the elevated peak beta power finding. The participants with TSC on a GABAergic medication (red dashed line, pink box plot, *n* = 42) showed significantly greater peak beta power than participants with TSC not on a GABAergic medication (solid red, *n* = 7), who were not significantly different from typically developing controls (gray, *n* = 49). (**D**) Among participants with TSC on GABAergic medication (left), those with high seizure severity (red, *n* = 12) showed the greatest peak beta power. Seizure severity and GABAergic medication use were independently associated with elevated peak beta power; there was no significant interaction

Cluster-based permutation testing revealed that, compared to age- and sex-matched typically developing controls, children with TSC showed significantly greater absolute and periodic power in the beta range in both frontal and posterior regions (Figs. 3 and 4a, Table S2; absolute: frontal 10.6–25.1 Hz, *p* = 0.011 and posterior 9.7–31.4 Hz, *p* = 0.001; periodic: frontal 10.6–26.1 Hz, *p* = 0.001 and posterior 11.2–27.0 Hz, *p* = 0.001). As expected based on prior literature in typically developing toddlers, the TD participants on the group level exhibited both a low (10–19 Hz) and high (20–29 Hz) beta peak [50], whereas the TSC group exhibited a single high amplitude beta peak around 20 Hz. In the gamma range, the TSC group showed diminished periodic power (frontal 34.5–49.7 Hz, *p* = 0.002, and posterior 34.3–50.8 Hz, *p* = 0.006). We observed no clear age-related patterns (Fig S2), and males and females showed similar power spectra (Fig S3) and no significant differences in peak beta frequency nor amplitude.

Based on these results, post-hoc analyses were conducted to compare beta power and peak beta frequency between groups. It was observed from individual waveforms (Fig S4) that peak beta frequency often occurred on the cusp between low beta (12–19 Hz) and high beta (20–29 Hz), around 20 Hz; thus, the decision was made to analyze peak beta frequency across the broad beta range (12–29 Hz). Both frontal and posterior peak broad beta frequency were lower for the TSC group than the TD group in paired t-tests (*t’*s > 3.8, *p*’s < 0.0005: TSC frontal M = 20.9 Hz, posterior M = 20.5; TD frontal M = 24.6, posterior M = 24.1). For comparison with other literature, we also tested high beta 20–29 Hz and found significantly lower peak beta frequency for TSC than TD children (*t’*s > 5.8, *p*’s < 5E-6: TSC frontal M = 23.7 Hz, posterior M = 23.9 Hz; TD frontal M = 26.7 Hz, posterior M = 27.1 Hz). Of note, within the TSC group, broad beta peak frequency showed trends of a positive correlation with Mullen NVDQ (frontal: *r* = 0.30, *p* = 0.05; posterior: *r* = 0.27, *p* = 0.07), and significant negative correlations with seizure severity (frontal: *r*=-0.34, *p* = 0.02; posterior: *r*=-0.31, *p* = 0.03).

*Association with cognition.* A previous study of young children with TSC had found associations between Mullen NVDQ scores and peak alpha frequency [15]. Though not a primary aim of this study, we explored this possible association in our data to test the replicability of this finding since replication opportunities are rare in rare genetic syndromes. In our sample, we observed no significant association between Mullen NVDQ and peak alpha frequency (frontal *r*=-0.21 *p* = 0.17; posterior *r*=-0.11 *p* = 0.48, uncorrected p values). Further research with larger samples with a narrower age range may clarify the association between peak alpha frequency and cognition in TSC.

*Aperiodic features.* The four most informative aperiodic variables (intercepts and slopes for frontal and posterior regions) were entered into a logistic regression model predicting group status (TD, TSC). The odds ratio generated from the logistic regression can be interpreted as the odds that an individual falls in the TSC group, rather than the TD group, given a 1-point change in modeled aperiodic intercept (i.e., from 0.01 to 1.01), which has a range in this sample of -0.04 to 1.00. In the model, posterior intercept reflecting broadband, nonrhythmic neuronal firing, emerged as a strong and sole significant predictor of group status (Fig. 3). Specifically, when holding frontal intercept, frontal slope, and posterior slope constant, a 1.0 point increase in posterior aperiodic intercept corresponded to a 222.9 increase in odds of belonging to the TSC group (OR = 222.9, *p* = 0.0037, 95% CI [7.2, 11,312.5]), corresponding to greater posterior broadband, nonrhymic neuronal firing in participants with TSC compared to matched controls. Aperiodic offset was not associated with GABA agonist use nor seizure severity (*p*’s > 0.2, *r*’s < 0.2). Of note, slope, which reflects the excitatory-inhibitory balance, did not predict group membership.

*Sensitivity analyses.* Results from all analyses remained consistent after excluding four participants with TSC who had brain surgery to improve their epilepsy, along with excluding their corresponding TD matches. Power spectra stratified by other potential variables of interest (age bins, sex, current seizures, infantile spasms) are depicted in the supplement (Figures S2, S3, S5, S6).

### Higher peak beta power is associated with both higher seizure severity, and GABA agonist use

Next, we examined the main effect of seizures on the power spectrum (Fig. 4a and b, S5, S6) for participants with TSC with available seizure severity data (*n* = 48 of 49). Participants with high seizure composite scores showed a marked increase in frontal peak beta power (*n* = 13; M(SD) = 0.52(0.17)) compared to those with moderate scores (*n* = 23; (M(SD) = 0.28(0.16)), low scores (*n* = 12; M(SD) = 0.24(0.12)), or typically developing controls (M(SD) = 0.20(0.09)). There was a significant main effect of seizure severity on frontal broad beta peak amplitude F(3, 90) = 29.28, *p* < 0.001, and post-hoc Tukey HSD tests showed a significant difference between all groups and the high seizure severity group (all adjusted p’s < 0.0005).

Since GABAergic medications are often used to manage epilepsy, we stratified the TSC sample by GABAergic medication use (Fig. 4c, Fig S7). Medication data were available for all participants, and only 7 of 49 TSC participants were not on a GABAergic medication. Despite the small sample, we observed a significant increase in beta amplitude in participants on GABAergic medications (*n* = 42; M(SD) = 0.38(0.19)) versus off (*n* = 7; M(SD) = 0.15(0.09)); individuals off GABAergic medication showed a beta peak more similar to their TD matches (M(SD) = 0.20(0.09)) than to other individuals with TSC. Specifically, there was a significant main effect of GABA agonist medication on frontal broad beta peak amplitude *F*(2, 90) = 16.42, *p* < 0.001), and post-hoc Tukey HSD tests showed a significant difference between all groups and the GABA agonist group (all adjusted *p*’s < 0.00005).

Finally, individuals with high seizure scores on GABAergic medications (*n* = 12) showed the most elevated beta peak (Fig. 4d). As GABA agonists are prescribed for elevated seizure activity, it was important to determine whether GABA and seizure activity were independently associated with increased beta power. Both GABA status and seizure severity were significantly, independently associated with peak beta amplitude when included in the same model, and the interaction was not significant (F(2, 90) = 2.20, *p* = 0.12; Fig S8).

## Discussion

In resting state EEG data collected from children with TSC aged 12–37 months and age- and sex-matched typically developing children, the TSC group showed markedly increased beta power that appeared driven by individuals with high seizure activity, as well as those on GABAergic antiepileptic medication. Seizure activity and GABAergic antiepileptic medication use are confounded by indication, so it is notable that there was no significant interaction between GABAergic medication use and seizure severity; in other words, seizure severity and GABAergic medication are both independently associated with increased beta power in the TSC group.

### Beta power differences in context of seizures and GABA agonist use

TSC participants showed a notable elevated beta peak in both spectral absolute power and periodic power after decomposition with SpecParam. The beta peak was observed consistently in both frontal and posterior regions around 20 Hz in broad beta (12–29 Hz) and around 24 Hz in high beta (20–29 Hz, excluding low beta peaks from the average), whereas typically developing controls showed a peak beta frequency closer to 24 Hz in broad beta and 27 Hz in high beta. Increased beta peak power was associated with both increased seizure severity and GABA agonist use in the TSC cohort. Increases in beta power with GABA agonist use are consistent with prior reports in both humans and animal models [27–34]. Thus, it is likely that GABAergic medication use in our sample could be driving the increased beta power and decreased peak beta frequency, at least in part. However, in the current sample, GABA agonists alone likely do not explain the increased beta power and lower peak beta frequency observed in the TSC cohort. Surprisingly, seizure severity – likely reflecting excessive excitatory signaling – also independently positively correlated with increased beta power.

Prior EEG analyses in TSC have suggested dysmaturity based on less complexity (reduced entropy) and higher regularity (increased Hurst exponent) ([13, 14]). It is also possible that beta alterations we observe in TSC reflect differences in brain maturation. In a large longitudinal normative sample of nearly 600 children, Wilkinson et al. (2024) recently reported nonlinear early developmental changes in both low and high beta peaks. More specifically, high beta peak amplitude and frequency both increase during the first year of life, peaking at 12 months, followed by a subsequent decrease in amplitude and downward shift in frequency. Thus, the high beta peak in TSC with higher amplitude and lower frequency could be interpreted as a sign of delayed or altered maturation. This hypothesis was supported by a post-hoc negative correlation of -0.32 (uncorrected *p* = 0.03) between the Mullen NVDQ and frontal broad beta power within the TSC group. In addition, qualitatively we observed expected low and high beta peaks in the TD group, but a single lower frequency/high amplitude beta peak in the TSC group. Further longitudinal analysis could provide more insight into whether the 20 Hz beta peak observed in TSC is related to the low versus high beta peaks observed during the first year of life in TD infants.

Finally, it remains possible that TSC itself is characterized by increased beta power and lower peak beta frequency. For comparison, Duplication 15q carries a similar EEG signature of markedly increased beta power and high beta peak frequency of 23 Hz reported in a sample with a broad age range (9 months to 14.5 years) [32]. Notably, epilepsy shows the opposite association in Dup15q compared to our TSC cohort, with epilepsy diagnosis predicting less high beta power in Dup15q [51], and greater seizure severity predicting more beta power in TSC. In Fragile X, unusually high beta power has also been observed at a peak frequency of 30 Hz in a cohort of 3–7 year olds [52], with beta peak amplitude and frequency decreasing with age suggesting that peak beta frequency may be an index of brain maturity [50]. In our current TSC cohort no age effects were observed. In addition, those with the lowest seizure severity or those without GABAergic medication exhibited periodic spectra more similar to TD participants, suggesting that these factors may have a greater impact on beta activity than TSC diagnosis alone.

Outside of the beta band, permutation cluster analysis of the power spectra showed differences in clusters that start at the upper edge of the high alpha range and extended into the gamma range. However, these differences are likely related to the large neighboring beta peak observed in TSC. The findings are consistent with Dickinson et al. (2019) who found no significant group differences in relative alpha power at 12, 24, or 36 months in a smaller longitudinal sample (*n* = 23 children with TSC and *n* = 20 controls). Dickinson et al. (2019) also identified a trending positive association between TSC 24-month peak alpha frequency and 36-month NVDQ verbal and nonverbal that only survived correction for multiple comparison in the posterior region. In contrast, in our cross-sectional sample, no significant association was observed between NVDQ and peak alpha frequency. Further research with larger samples with a narrower age range may clarify the association between peak alpha frequency and cognition in TSC.

### Aperiodic power differences

Analysis of aperiodic power components demonstrated differences in aperiodic offset, but not slope. Furthermore, differences in aperiodic offset were only observed in posterior, but not frontal electrodes; posterior aperiodic offset showed no association with GABA agonist use nor seizure severity. Aperiodic offset is thought to reflect broad band firing of the network and increases in children with TSC could reflect differences in synaptic pruning or differences in maturational processes. Perhaps surprisingly we did not observe any differences in aperiodic slope between groups. As aperiodic slope is hypothesized to reflect E/I balance, one might expect children with TSC to have a flattened, reduced slope, reflecting increased excitation. Lack of differences may reflect countering effects of anti-seizure medications as well as our limited sample size.

### Resting EEG as a biomarker in the context of seizures and medication use

EEG data for this study was collected as the baseline timepoint of an RCT of the behavioral intervention JASPER [43]. At the start of the RCT, we hypothesized that resting EEG spectral power might serve as a biomarker - either as an outcome measure, or to predict treatment response. These prospects could be undermined by the associations of increased beta oscillatory activity with high seizure activity and some medications (i.e., some antiepileptics, some anxiolytics). It is neither feasible nor ethical to control this confound by requiring participants with epilepsy to stop medication use during a behavioral intervention trial such as our JASPER RCT, as medications often provide critical control of seizures. This challenge is true for not only TSC, but also all neurodevelopmental conditions with epilepsy. This challenge also extends to conditions with co-occurring anxiety for which benzodiazepines – GABA agonists known to increase beta power – are prescribed. However, neural markers of intervention response are desperately needed, and EEG features offer great promise as EEG is tolerated more readily than MRI. To address this issue, one solution might be to avoid resting EEG biomarkers in the beta band. Another solution might entail requiring candidate resting EEG biomarkers to show a limited group-level correlation at baseline with medication status or seizure severity, before proceeding with individual-level analyses of change over time. This approach would demonstrate that the candidate biomarkers are less susceptible to medication status and seizure severity during the course of the RCT. Accordingly, we recommend that future studies of resting EEG in neurodevelopmental conditions of all ages collect comprehensive seizure and GABAergic medication data at each EEG data collection point, including changes in medications, so that final analyses can account for exogenous influences on candidate biomarkers. Another recommendation is that ideally, researchers collect longitudinal EEG data, beginning prior to the start of seizure medications. While age-related changes would need to be considered, neurophysiological features could be compared before and after medication start. Finally, distinguishing the effects of medications, seizures, and development on EEG features requires large, heterogenous samples. Recruiting large numbers of children with rare genetic disorders can be quite challenging, but advances in early diagnostics and increased funding for research in this area would support the recruitment of more robust samples.

Limitations of this analysis include its cross-sectional design across a broad age range (12–37 months), absence of tuber location MRI data, small sample size of seizure severity and medication subsamples, and reliance on parent-report for medication, seizure, and TSC diagnosis.

## Conclusions

This paper adds to the current literature on resting EEG spectral power biomarkers in neurodevelopmental disorders by identifying common confounding variables that impact beta power (seizures, antiepileptic GABAergic medication use). To the TSC literature specifically, this paper contributes a characterization of resting EEG in toddlers, benchmarked against typical development. Through this careful comparison, we were able to identify elevated beta power in children with TSC, and relate it to sample characteristics (medication use, increased seizure severity). Future directions include investigation of identified resting EEG features in relation to RCT behavioral outcomes, and collection of larger samples of resting EEG in individuals with TSC to characterize beta power and peak beta in subgroups of medication use and seizure severity.

## Electronic supplementary material

Below is the link to the electronic supplementary material.

Supplementary Material 1

## Data Availability

The datasets used and/or analyzed during the current study are available from the corresponding author on reasonable request.

## Abbreviations

BEAPP: Batch Electroencephalography Automated Processing Platform
DQ: Developmental Quotient
Dup15q: Duplication 15q
EEG: Electroencephalography
ICA: Independent Component Analysis
ISP2: Infant Screening Project 2
JASPER: Joint Attention, Symbolic Play, Engagement & Regulation
JETS: JASPER Early Intervention for Tuberous Sclerosis Complex Study
mTOR: Mammalian Target of Rapamycin
MSEL: Mullen Scales of Early Learning
MARA: Multiple Artifact Rejection Algorithm
NVDQ: Nonverbal Developmental Quotient
RCT: Randomized Controlled Trial
SD: Standard Deviation
TAND: TSC-Associated Neuropsychiatric Disorders
TD: Typically Developing
TSC: Tuberous Sclerosis Complex
