# Supplementary material for: Resting state EEG in young children with Tuberous Sclerosis Complex: associations with medications and seizures
Source: J Neurodev Disord. 2025 Jan 18;17:2. doi: 10.1186/s11689-025-09590-z (PMC11742757; doi:10.1186/s11689-025-09590-z)
Supplement: Supplementary file 1 — Supplementary Material 1 [file 11689_2025_9590_MOESM1_ESM.docx]

# SUPPLEMENT

Table S1. *GABAergic Medications*

Table S2. Significantly different clusters across frequency bands

Figure S1. Electrode map depicting regions of interest

Figure S2. Group differences in frontal power spectrum binned by age

Figure S3. Power spectrum stratified by sex

Figure S4. Individual frontal broad beta peaks

Figure S5. Power spectrum stratified by whether individual experienced any seizure(s) in the last 2 months

Figure S6. Power spectrum stratified by infantile spasms

Figure S7. Power spectrum stratified by GABA agonist use

Figure S8. Individual power spectra stratified by age, GABA agonist use, and whether individual experienced any seizure(s) in the last 2 months

#### Table S1

Table S1. *GABAergic Medications*

| **Generic Medication (Brand)** | **GABA agonist** |
| --- | --- |
| Brivaracetam (Briviact) | No |
| Everolimus (Afinitor) | No |
| Lacosamide (Vimpat) | No |
| Lamotrigine (Lamictal) | No |
| Levetiracetam (Keppra) | No |
| Oxcarbazepine (Trileptal) | No |
| Perampanel (Fycompa) | No |
| Sirolimus (Rapamune) | No |
| Zonisamide (Zonegran) | No |
| Clobazam (Onfi) | Yes |
| Felbamate (Felbatol) | Yes |
| Phenobarbital (Luminal) | Yes |
| Topiramate (Topamax) | Yes |
| Valproic Acid, Sodium Valproate | Yes |
| Vigabatrin (Sabril; Vigadrone) | Yes |
| Cannabidiol (Epidiolex) | Unknown^1^ |

*Note.* The mechanism of action of Cannabidiol relative to GABA is unknown. All participants who taking cannabidiol were also taking a GABA agonist.

#### Table S2

Table S2. Significantly different clusters across frequency bands

| **Canonical frequency band** | **Significant clusters (Hz)** | **p-values** | **Canonical Frequency Band** |
| --- | --- | --- | --- |
| **Frontal** |  |  |  |
| Absolute | 10.6-25.1 | 0.011 | Alpha, beta |
| Periodic | 10.6-26.1 | 0.001 | Alpha, beta |
|  | 34.5-49.7 | 0.003 | Gamma |
| **Posterior** |  |  |  |
| Absolute | 9.7-31.4 | 0.002 | Alpha, beta, gamma |
| Periodic | 11.2-27.0 | 0.001 | Alpha, beta |
|  | 34.3-50.8 | 0.006 | Gamma |

#### Figure S1

Supplemental Figure 1. Electrode map depicting frontal (red) and posterior (blue) regions of interest


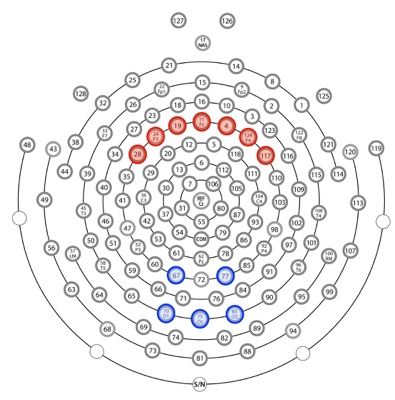


#### Figure S2

Supplemental Figure 2. Group differences in frontal power spectrum binned by age (frontal on left, posterior on right). Top plots depict absolute power and bottom plots depict periodic, oscillatory power (*i.e.*, absolute power minus modeled 1/f^X^ decay curve).

Frontal Posterior


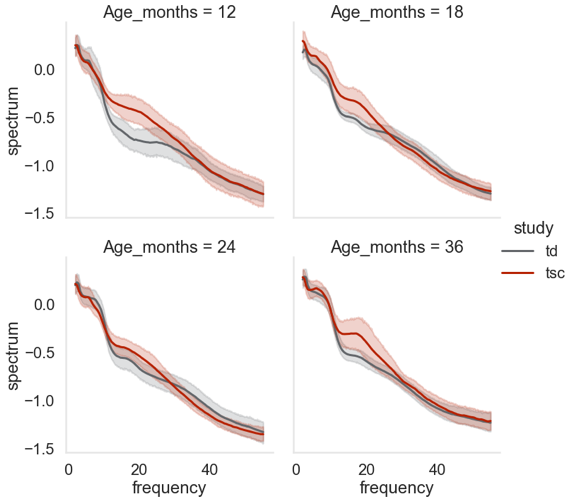

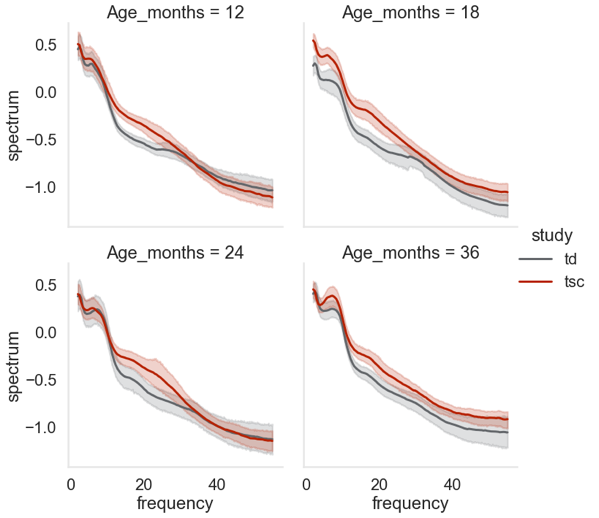


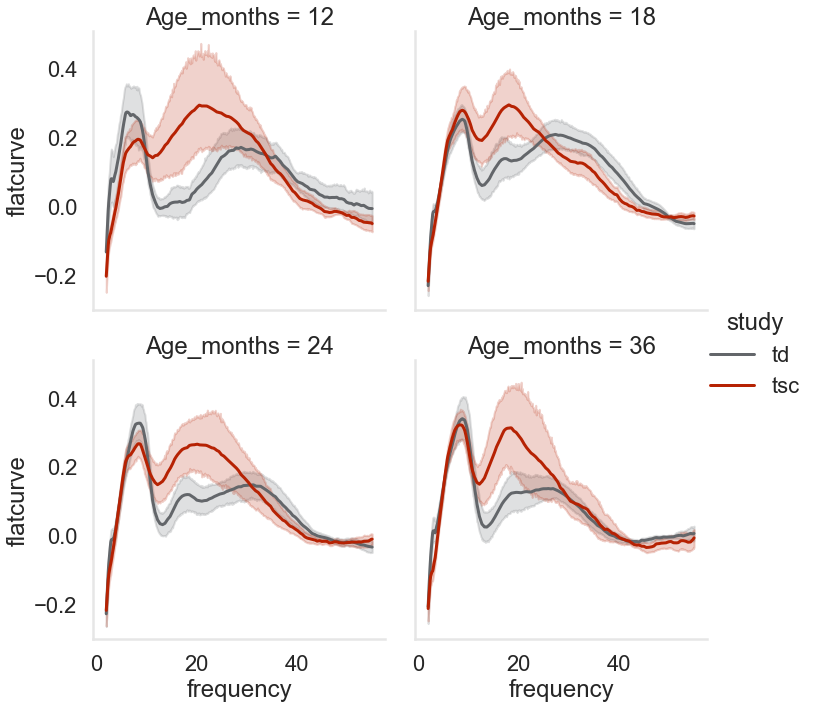

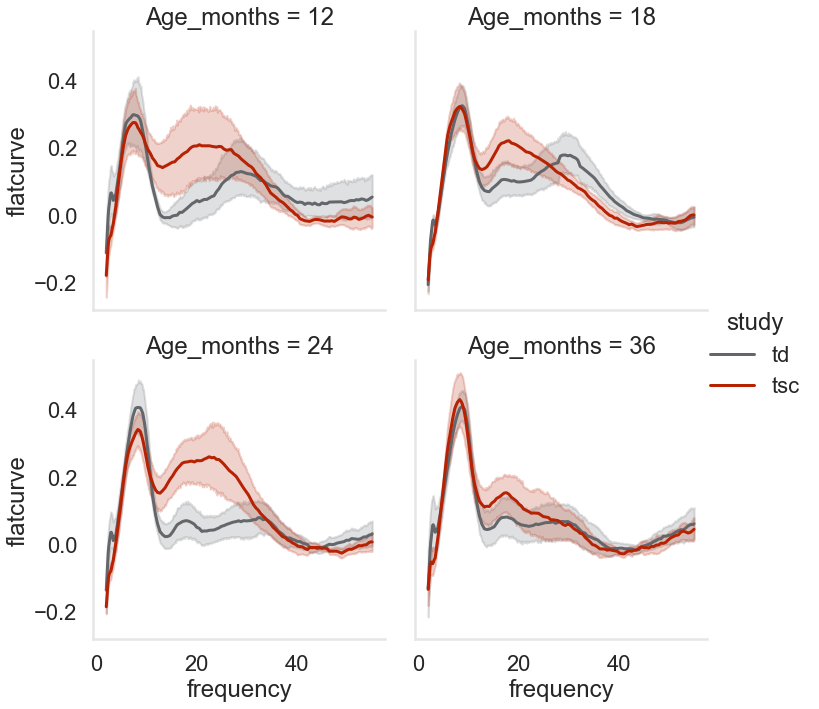


#### Figure S3

Supplemental Figure 3. Power spectrum stratified by sex (frontal on top, posterior on bottom). Top plots depict absolute power and bottom plots depict periodic, oscillatory power (*i.e.*, absolute power minus modeled 1/f^X^ decay curve).

Frontal

TD TSC


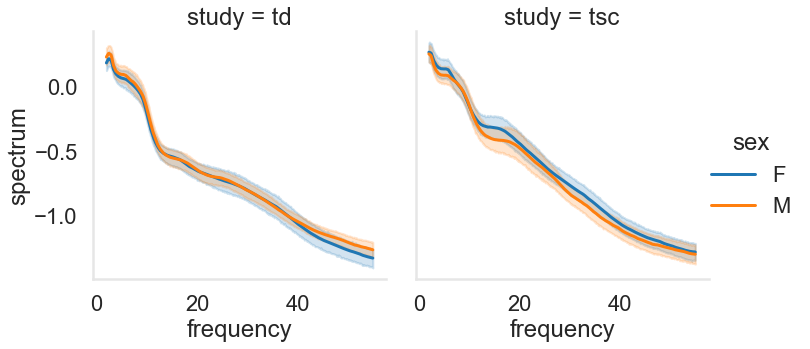


Posterior

TD TSC


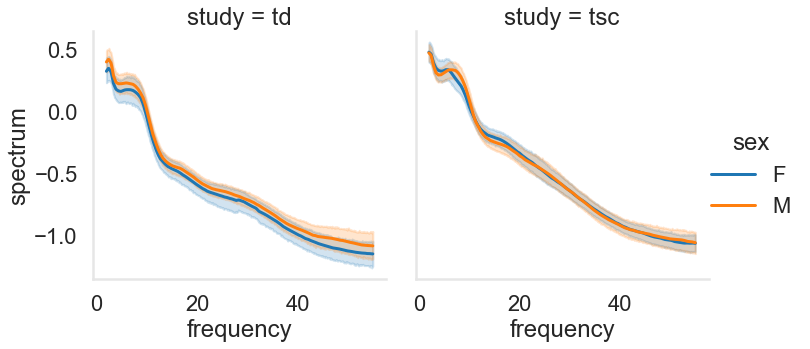


#### Figure S4

Supplemental Figure 4. Individual frontal broad beta peaks between 12 and 29Hz identified in the TSC cohort (green, top) and the TD cohort (red, bottom)


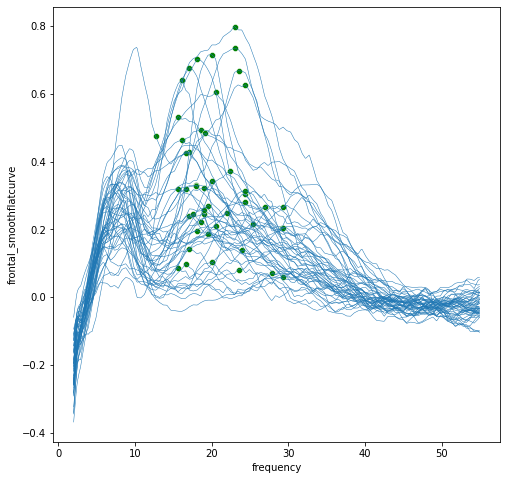
TSC


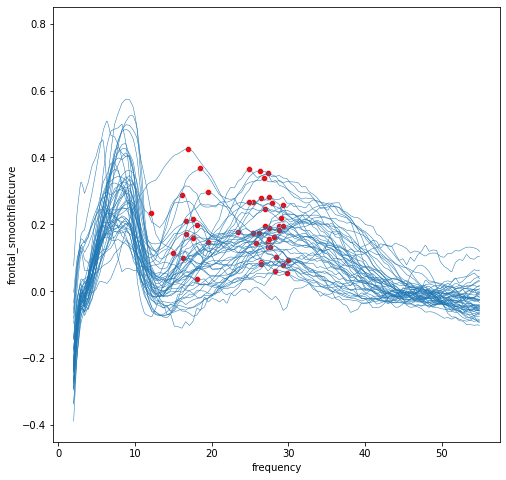
TD

#### Figure S5

Supplemental Figure 5. Power spectrum stratified by whether individual experienced any seizure(s) in the last 2 months, per parent report (frontal on left with legend, posterior on right). Top plots depict absolute power and bottom plots depict periodic, oscillatory power (*i.e.*, absolute power minus modeled 1/f^X^ decay curve).

Frontal Posterior


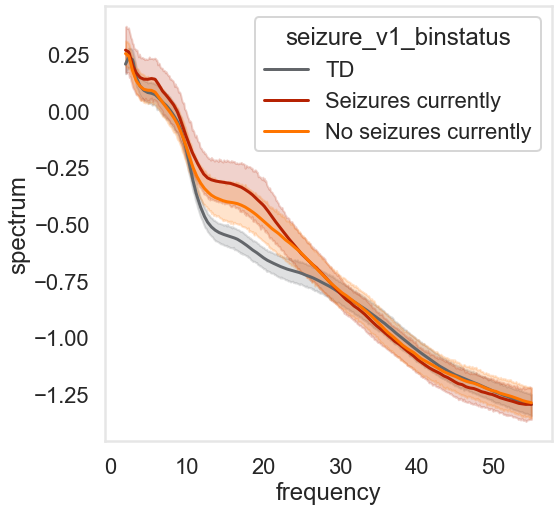

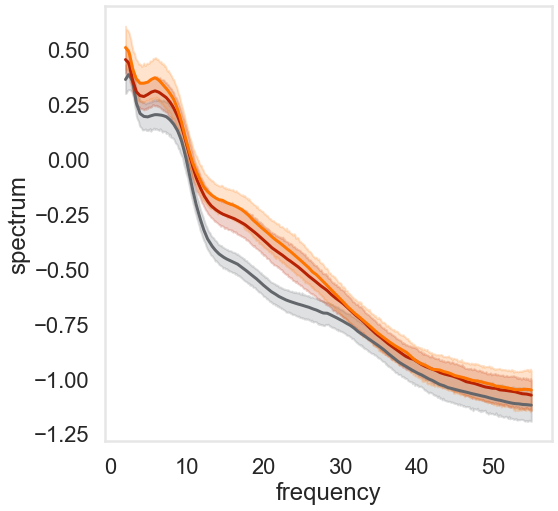


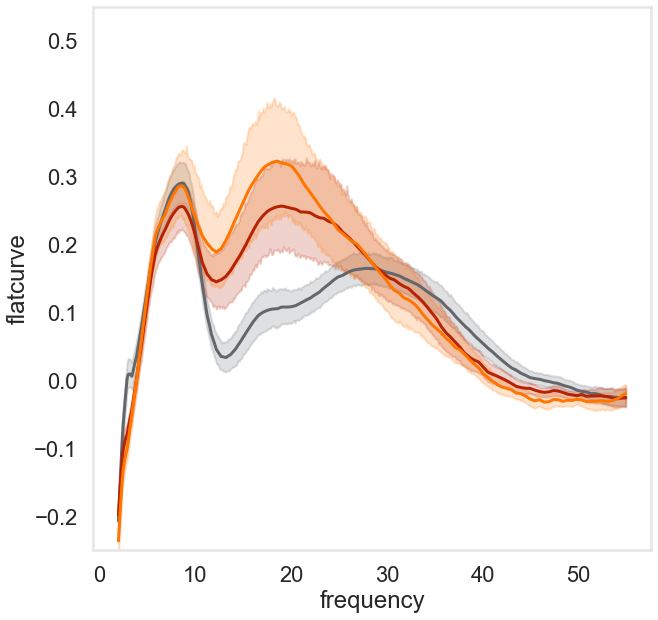

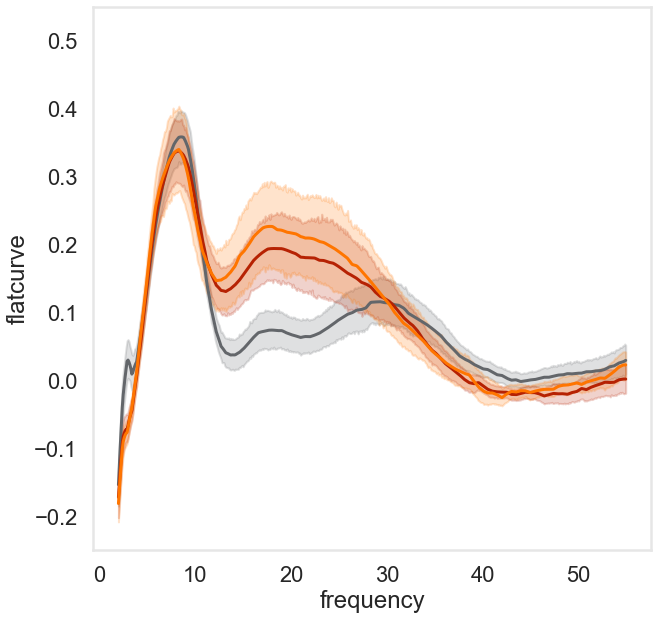


#### Figure S6

Supplemental Figure 4. Power spectrum stratified by infantile spasms, per parent report (frontal on left with legend, posterior on right). Top plots depict absolute power and bottom plots depict periodic, oscillatory power (*i.e.*, absolute power minus modeled 1/f^X^ decay curve).

Frontal Posterior


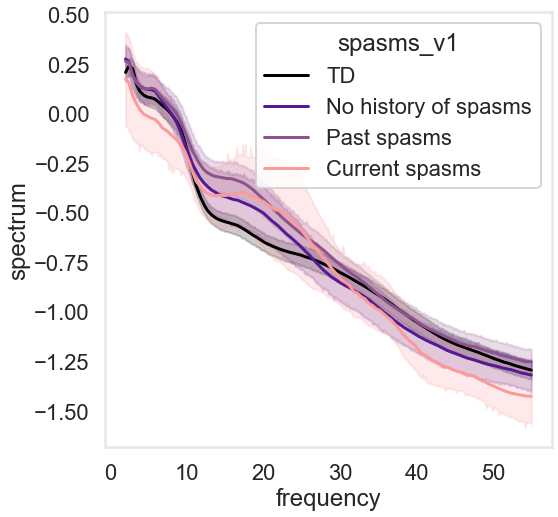

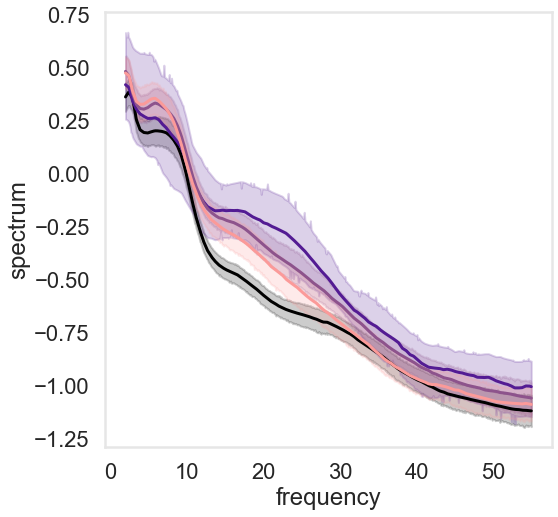


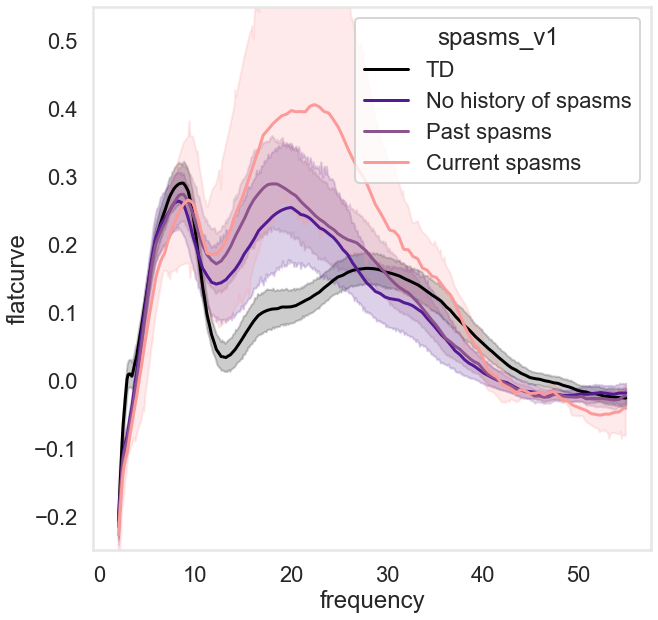

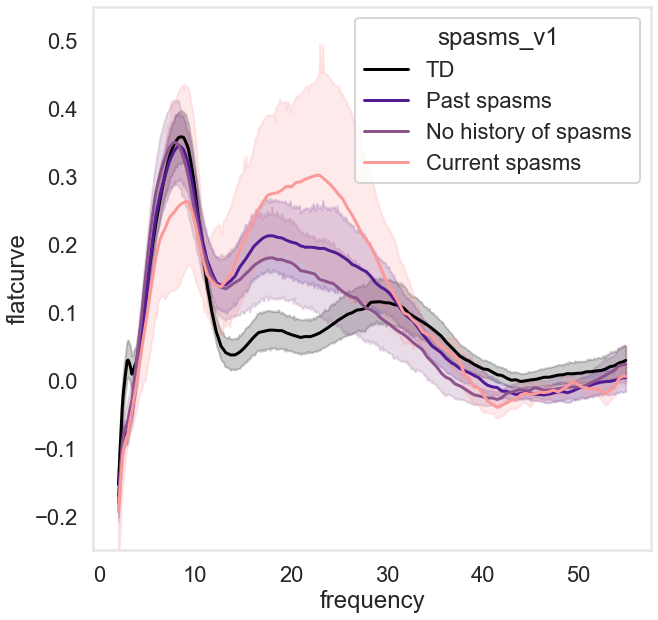


#### Figure S7

Supplemental Figure 7. Power spectrum stratified by GABA agonist use (frontal on left with legend, posterior on right). Top plots depict absolute power and bottom plots depict periodic, oscillatory power (*i.e.*, absolute power minus modeled 1/f^X^ decay curve).

Frontal Posterior


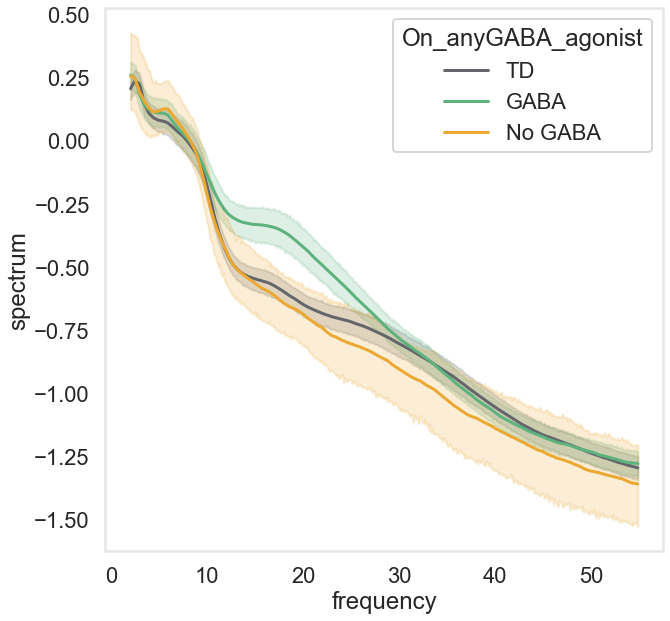

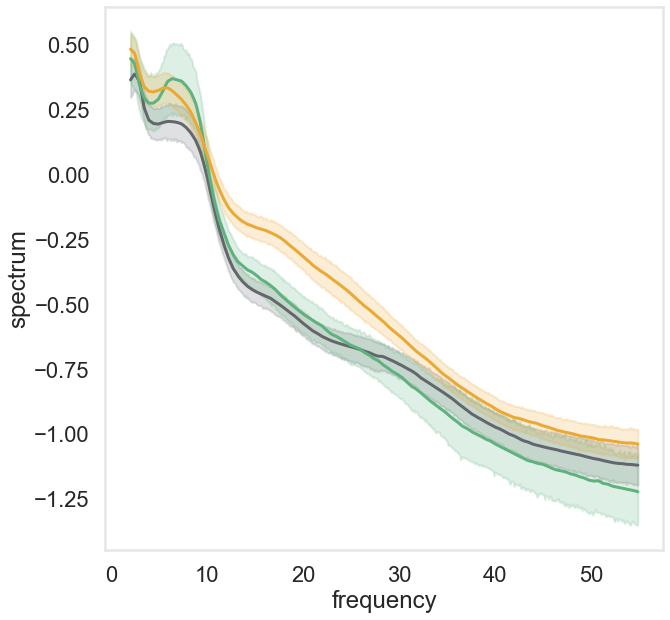


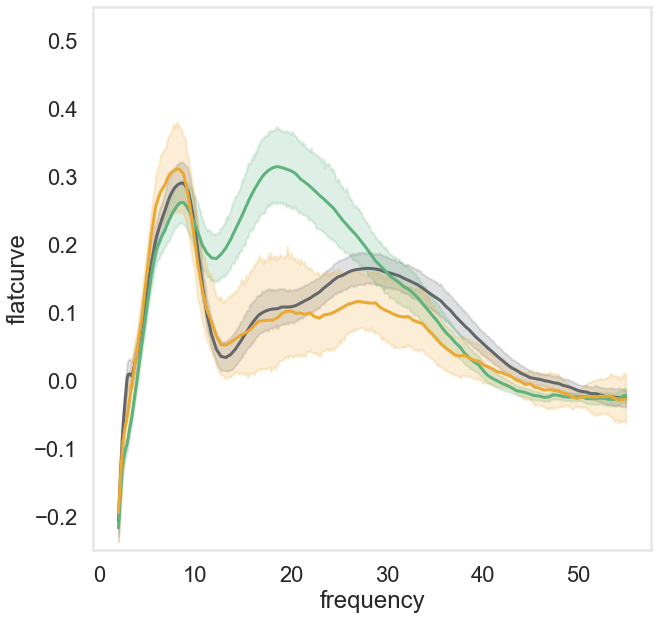

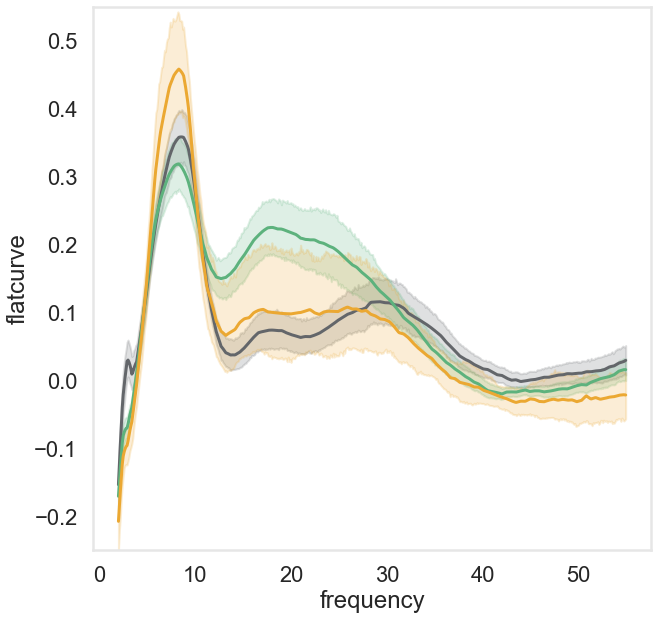


#### Figure S8

Supplemental Figure 6. Individual power spectra stratified by age, GABA agonist use, and whether individual experienced any seizure(s) in the last 2 months, per parent report. Top plots depict absolute power and bottom plots depict periodic, oscillatory power (*i.e.*, absolute power minus modeled 1/f^X^ decay curve). Groups: Has seizures, no GABA agonist (red, n=3); Has seizures, with GABA agonist (green, n=17); No seizures, no GABA agonist (blue, n=4); No seizures, with GABA agonist (yellow, n=25)


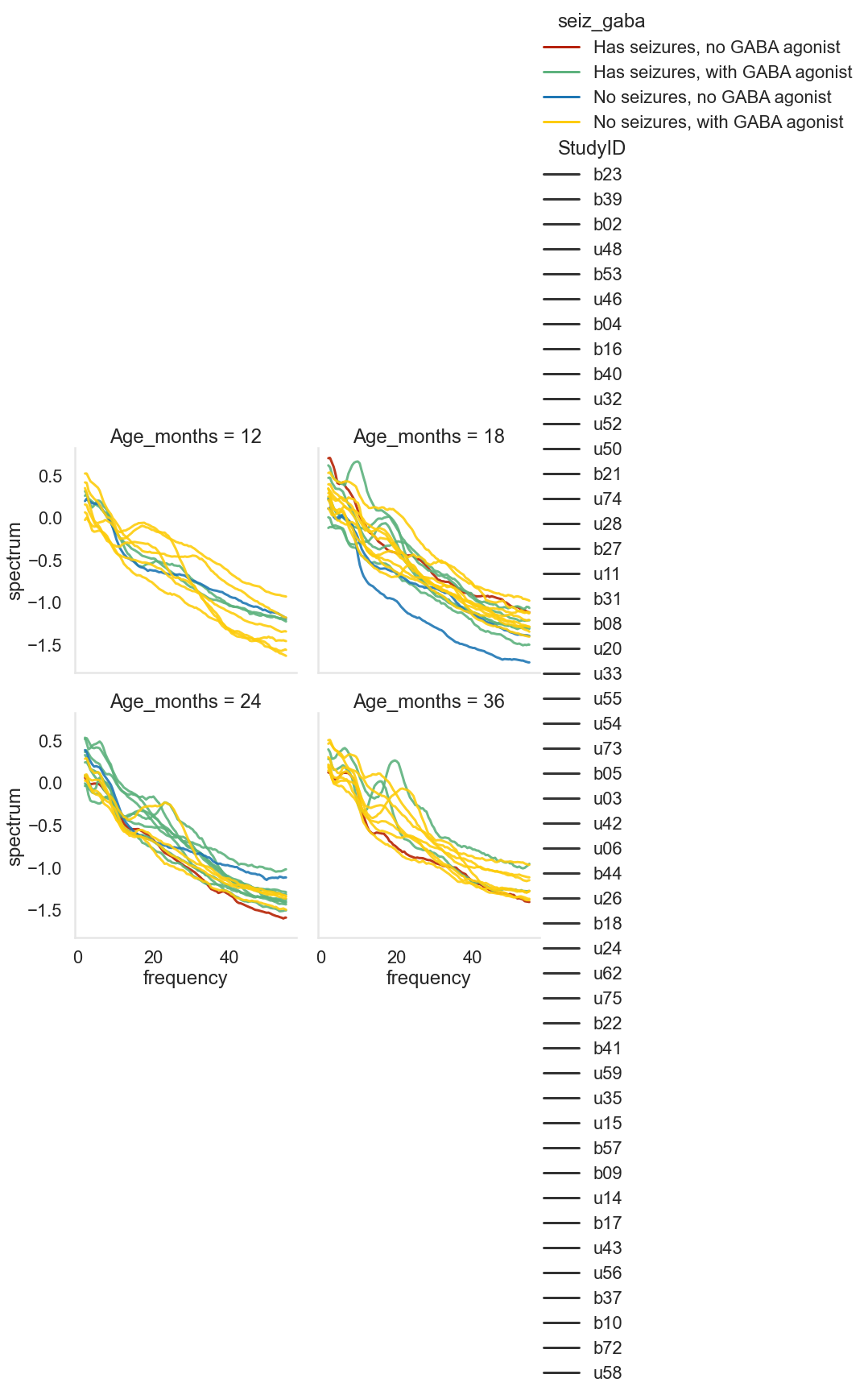

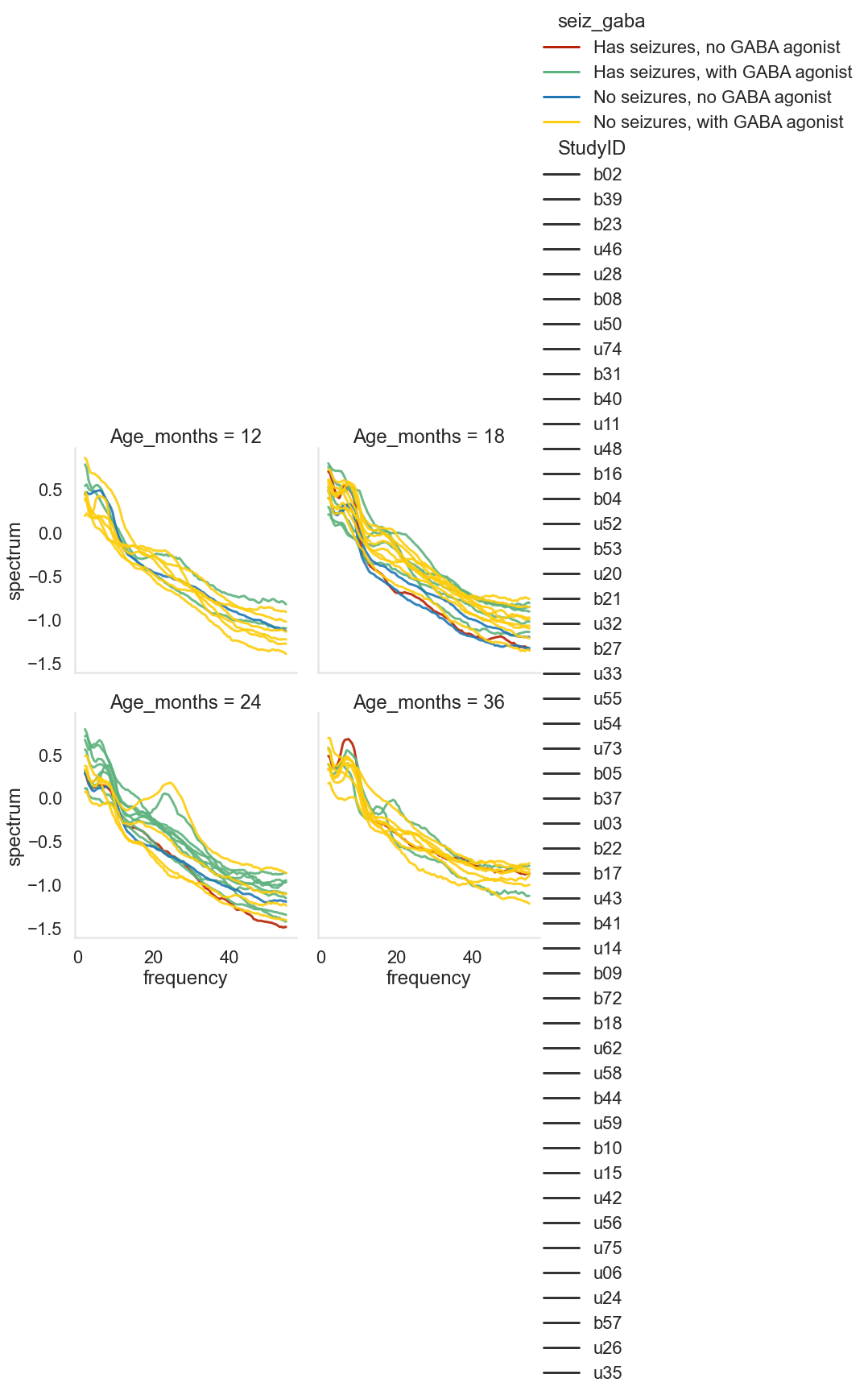

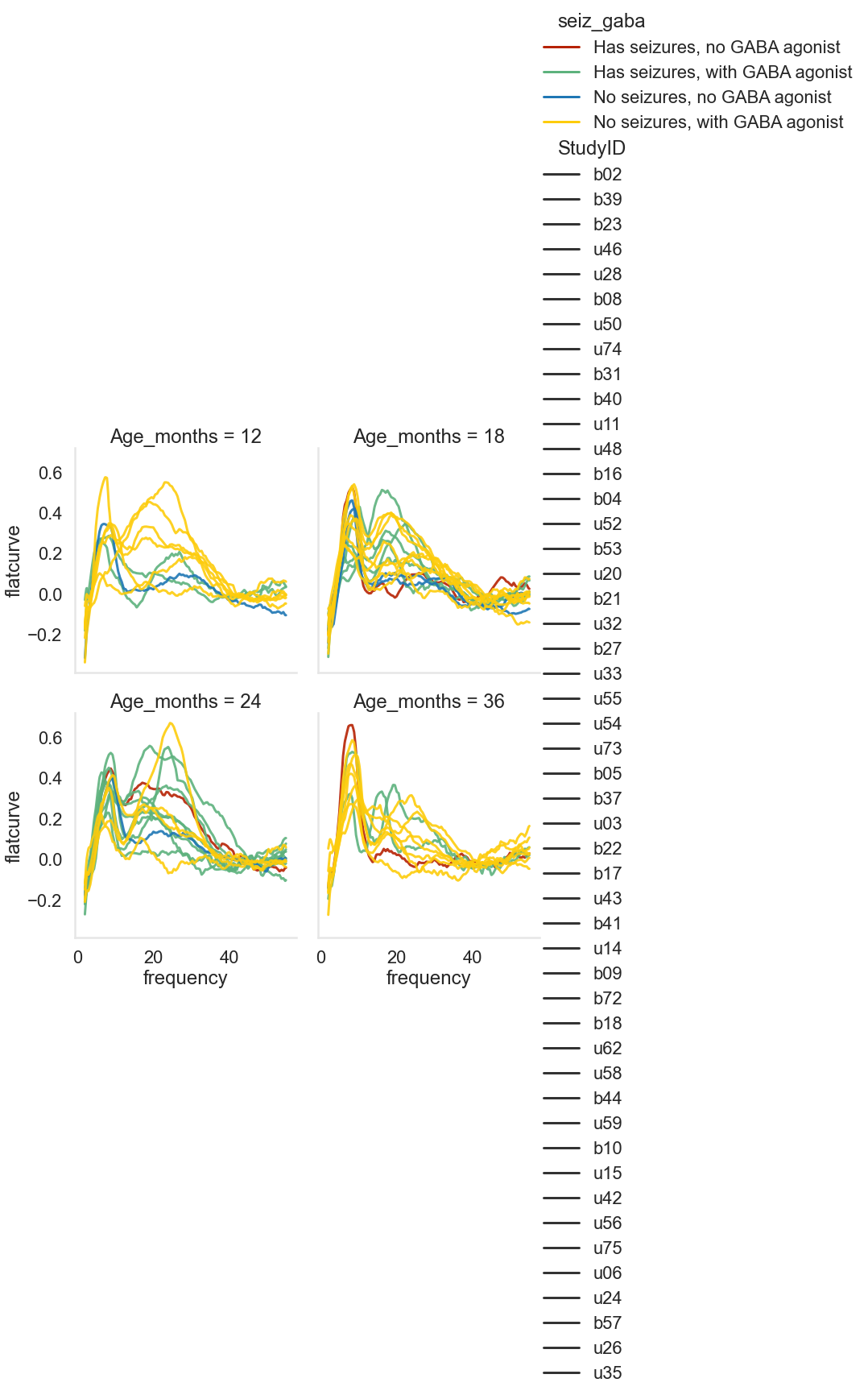

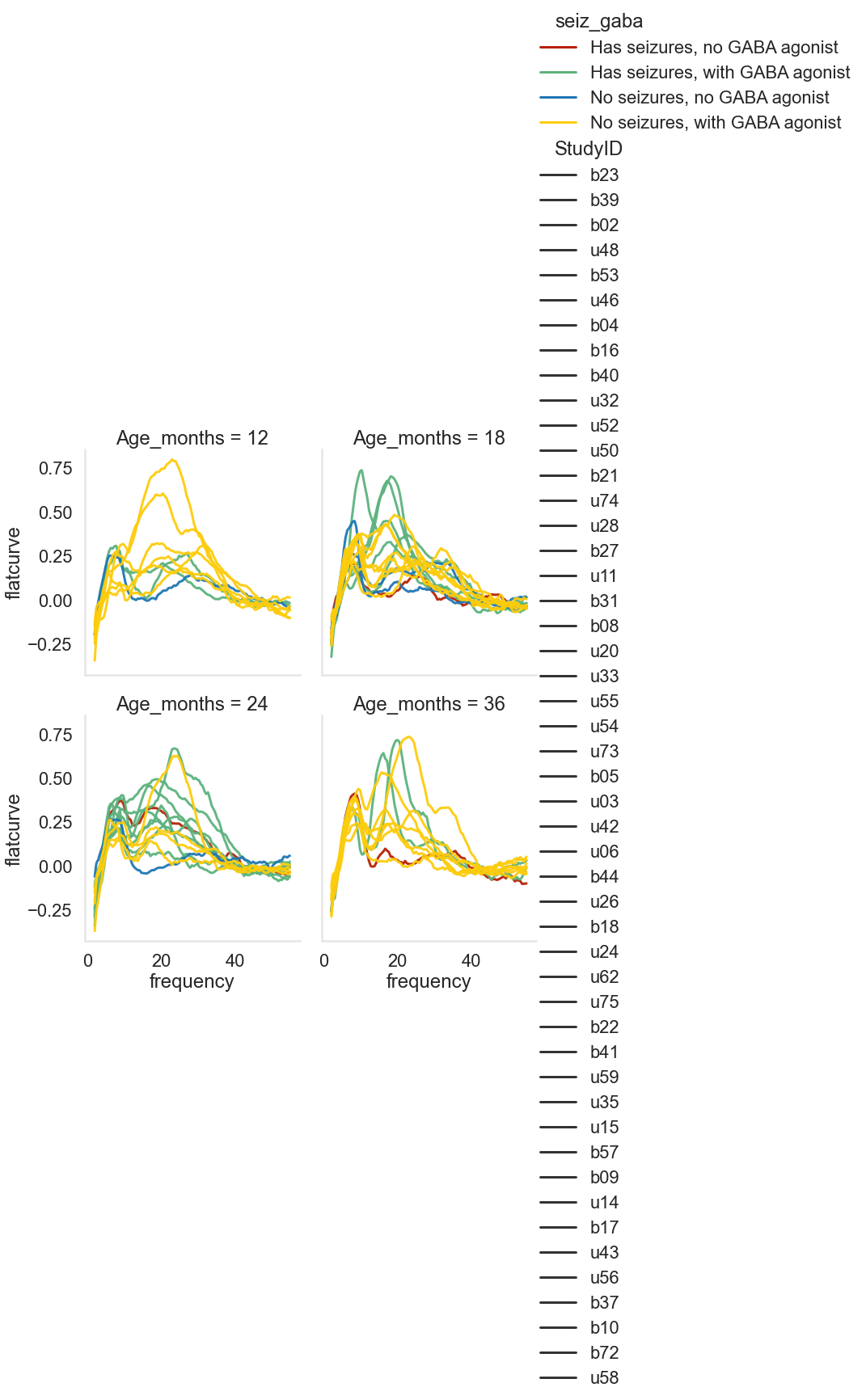

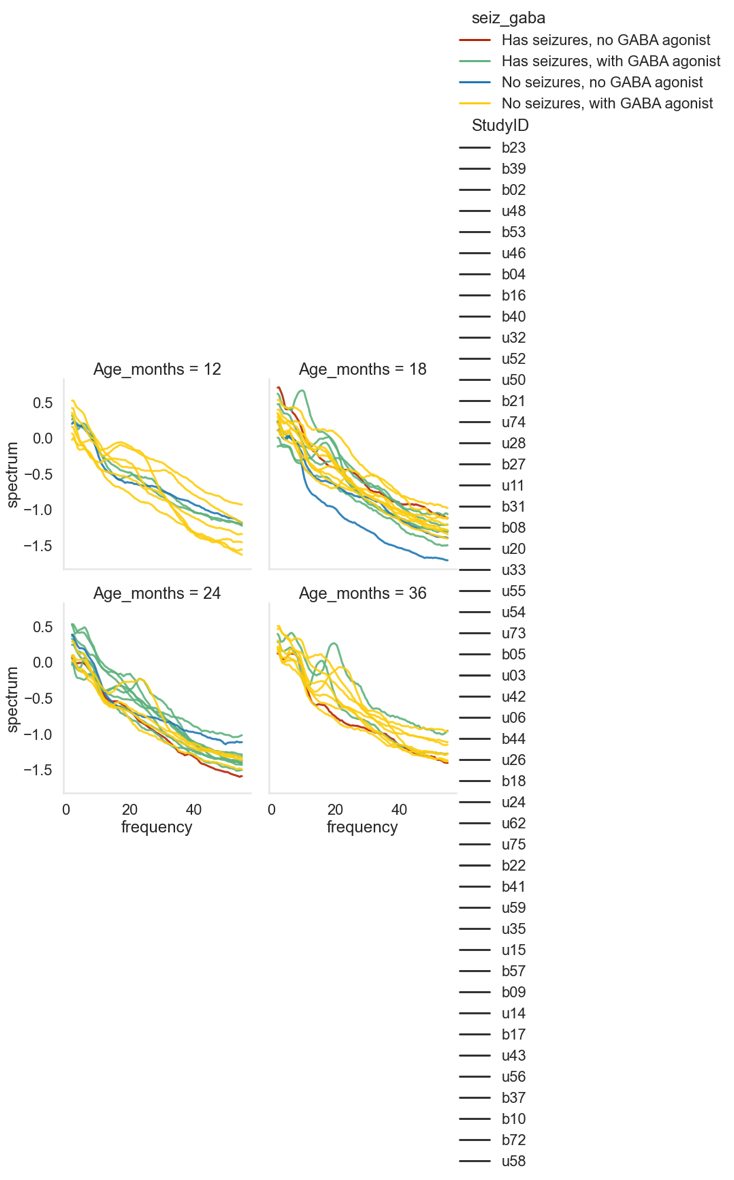


Frontal
